# Efficacy and safety associated with the infusion speed of intravenous immunoglobulin for the treatment of Kawasaki disease: a randomized controlled trial

**DOI:** 10.1186/s12969-021-00601-6

**Published:** 2021-07-03

**Authors:** Saori Fukui, Mitsuru Seki, Takaomi Minami, Kazuhiko Kotani, Kensuke Oka, Akiko Yokomizo, Daisuke Matsubara, Tomoyuki Sato, Yasuyuki Nozaki, Mari Saito, Yutaka Kikuchi, Kenji Miyamoto, Yukifumi Monden, Takanori Yamagata

**Affiliations:** 1 Department of Pediatrics, Jichi Medical University, 3311-1, Yakushiji, Shimotsuke, Tochigi, 329-0498 Japan; 2 Department of Pediatrics, Shin-Oyama City Hospital, Tochigi, Japan; 3 Division of Community and Family Medicine, Jichi Medical University, Tochigi, Japan; 4 Department of Pediatrics, Haga Red-Cross Hospital, Tochigi, Japan; 5 Department of Pediatrics, Dokkyo Medical University, Tochigi, Japan; 6 Department of Pediatrics, International University of Health and Welfare Hospital, Tochigi, Japan

**Keywords:** Kawasaki disease, Intravenous immunoglobulin, IVIG, Randomized study, Safety, Effectiveness

## Abstract

**Background:**

High-dose intravenous immunoglobulin (IVIG) is the mainstay of treatment for Kawasaki disease (KD). Usually, 2 g/kg of IVIG is administered over 10–24 h, depending on the institution or physician, but the association between infusion speed and effectiveness has not been reported. In this study, we evaluated the differences in efficacy and safety between two different IVIG administration speeds.

**Methods:**

This was a multicenter, unblinded, randomized controlled study. Patients newly diagnosed with KD were randomized into two groups: one who received IVIG over 12 h (12H group, double speed), and one that received IVIG over 24 h (24H group, reference speed). The endpoints included the duration of fever, incidence of coronary artery abnormalities (CAAs) and of adverse events. Laboratory data were evaluated before and after IVIG administration.

**Results:**

A total of 39 patients were enrolled. There was no difference between groups in fever duration after the initiation of IVIG (21 h vs. 21.5 h, *p* = 0.325), and no patient experienced CAAs. Two adverse events were observed in the 12H group (elevation of aspartate aminotransferase and vomiting), however no severe adverse events requiring treatments or extension of hospital stay were observed in either group. After initial IVIG administration, the change ratio of inflammatory markers, such as white blood cell counts, neutrophils, C-reactive protein, and albumin, did not show significant differences between the two groups. On the other hand, a greater increase of serum immunoglobulin G from its baseline level was observed in the 24H group compared to the 12H group (3037 ± 648 mg/dl vs. 2414 ± 248 mg/dl, *p* < 0.01).

**Conclusion:**

The efficacy and safety of IVIG administered over 12 h (double speed) were similar to those administered over 24 h (reference speed).

**Trial registration:**

University Hospital Medical Information Network (UMIN000014665). Registered 27 July 2014 – Prospectively registered, https://upload.umin.ac.jp/cgi-open-bin/ctr/ctr_view.cgi?recptno=R000017058

## Background

Kawasaki disease (KD) is an acute, self-limited, systematic vasculitis of unknown cause that occurs predominantly in infants and young children. KD is markedly more prevalent in Japan, where 264.8/1,000,000 children aged under 5 years were treated in 2012 [1]. The most important complication of KD is coronary artery abnormalities (CAAs), which occur in approximately 25% of untreated patients [2]. Since high-dose intravenous immunoglobulin (IVIG) can greatly reduce the incidence of CAAs [3], high-dose IVIG plus aspirin is the mainstay of treatment in the acute phase of KD. In 1991, Newburger et al. reported that a single infusion of immunoglobulin at 2 g/kg was more effective than daily infusions of 400 mg/kg for four consecutive days, with a lower incidence of CAAs [4]. In addition, meta-analysis revealed that 2 g/kg in a single infusion showed superiority over 400 mg/kg/day for 5 days in the duration of fever in acute phase and the reduction of CAAs [3]. Based on these observations, a single infusion of IVIG 2 g/kg was established as the primary treatment in the acute phase of KD.

According to the American Heart Association guidelines, 2 g/kg of IVIG is usually administered over 10–12 h [5]. On the other hand, infusions of 2 g/kg IVIG over both 12 and 24 h are used in Japan, depending on the institution or physician. Although the short dosage of IVIG administration can have the advantage of reducing inflammation quickly and allowing for earlier decisions on additional treatment, there are concerns about the adverse effects of administrating large amounts of IVIG in a short period of time, for instance, headache, skin rash, vomiting, thrombosis, or shock. Since no study has compared the efficacy and safety of different infusion speeds of IVIG, we performed a randomized clinical trial to ascertain whether there are differences in the efficacy and safety between IVIG administered over 12 h and 24 h.

## Methods

### Patients

We enrolled patients between December 2014 and January 2019 at five centers in Japan. Patients aged < 15 years who were newly diagnosed with KD between 4 and 7 days of illness (day 1 was defined as the first day of fever) were enrolled. Eligible patients also had at least five of the six major symptoms according to the definition outlined in the Japanese diagnostic guidelines for KD [6]. The major exclusion criteria were: (1) patients without a fever at the time of enrollment, (2) patients with serious bacterial infections, (3) patients with CAAs at the time of enrollment, (4) patients who had received systemic steroids within the previous 4 weeks, and (5) patients with underlying diseases, such as chromosomal abnormalities. We performed a sample size calculation based on a preliminary study. The mean difference in C-reactive protein after IVIG administration between the 12-h infusion group and 24-h infusion groups was approximately 2. Assuming that the standard deviation is 3, α = 5%, and β = 20%, we calculated a required sample size of 35. The target sample size was set at 40 patients, taking into account cases dropout after enrollment.

All patients or their legal guardians received adequate information using an informed consent form, and written informed consent was obtained from all legal guardians of the patients before enrollment. This study was approved by the institutional review boards of all participating institutions (number CB13–49). This trial was registered with the University Hospital Medical Information Network (UMIN no. 000014665).

### Study protocol

Participating patients were randomly allocated to either a 12 h (12H group) or 24 h (24H group) IVIG administration group, with stratification according to age and sex. All patients in both groups received initial IVIG treatment (Venilon-I, Teijin Pharma Limited, Tokyo, Japan) at a dosage of 2 g/kg, and aspirin 30 mg/kg per day. The patients allocated to the 12H group were treated with IVIG infusion over the course of 12 h, while those in the 24H group were treated over the course of 24 h. Aspirin was reduced to 5 mg/kg per day after defervescence for two consecutive days.

Patients’ axillary temperature was obtained every 6 h until 7 days after the initial IVIG treatment. Patients were regarded as afebrile when their axillary temperature dropped below 37.5 °C for at least 48 h, and the start point of 48 h was defined as the time of defervescence.

When a patient’ s axillary temperature was greater than 37.5 °C at 48 h after the initiation of the initial IVIG treatment, they were defined as IVIG resistant and an additional dose of IVIG 2 g/kg was administered as a second-line treatment at the same infusion speed as the initial IVIG. Relapse was defined in cases of recurrent fever, despite defervescence after the initial IVIG treatment. Patients who had persistent fever after the first and second IVIG treatments received a third-line treatment according to the treatment protocol of each institution.

The risk score of IVIG non-responders was calculated using the risk scoring system by Kobayashi et al., which consists of 11 points based on five blood examinations and two patient characteristics [7]. Laboratory testing (white blood cell count, percentage of neutrophils, platelet count, aspartate aminotransferase, sodium, C-reactive protein, and serum level of immunoglobulin G (IgG)) was performed at the time of enrollment (study day 0), 48 h after initial IVIG (day 2), and 7 days after initial IVIG (day 7). Echocardiograms were obtained at each institution on day 0 (before the initial treatment) and on days 2, 7, and 30 after the initial IVIG administration. The absolute diameters of the right coronary artery, left main coronary artery, left anterior descending artery, and left circumflex artery were measured. Patients were diagnosed with CAAs according to the Japanese Ministry of Health criteria, which is based on absolute values (> 3 mm in children < 5 years old and > 4 mm in children ≥5 years old) or relative increase (≥1.5 times greater than adjacent segments) of the internal diameter of the coronary arteries. Echocardiography was performed by pediatricians at each facility. Patients, treatment physicians, and echocardiography assessments were not blinded to the assignment. An adverse event was defined as any unintended clinical symptoms or abnormal laboratory values observed after initial IVIG administration: fever, headache, skin rash, nausea, vomiting, neutropenia, liver dysfunction, renal dysfunction, lung edema, heart failure, shock, meningitis, and thrombosis. A severe adverse event was defined as any requiring additional treatment or extension of hospital stay.

### Outcomes

The primary outcomes were (1) the median duration of fever after the initiation of IVIG and (2) the cumulative risk of CAAs. The secondary outcomes were as follows: (1) the risk of additional IVIG treatment, total dose of IVIG treatment, and risk of third-line treatment; (2) laboratory data changes at days 0, 2, and 7; and (3) adverse events.

### Statistical analysis

Statistical analyses were conducted using Fisher’s exact test or χ^2^ test (categorical data) and the Wilcoxon–Mann–Whitney test (continuous data). Repeated measures ANOVA were used to analyze changes in the laboratory data. A 2-sided *P* value < 0.05 was considered statistically significant. All statistical analyses were performed using EZR (Saitama Medical Center, Jichi Medical University, Saitama, Japan), which is a graphical user interface for R version 4.0.0 (The R Foundation for Statistical Computing, Vienna, Austria). More precisely, it is a modified version of R commander designed to add statistical functions frequently used in biostatistics [8].

## Results

### Patient characteristics

During the study period, 39 eligible patients were enrolled and randomized. Nineteen patients were assigned to the 12H group, and 20 were assigned to the 24H group. One patient in the 12H group presented with a small CAA at enrollment, which was revealed retrospectively. This patient was included in this study. The patient characteristics are shown in Table 1. Age, sex, and the day of initial IVIG treatment did not differ between the two groups. Regarding laboratory findings, serum concentrations of sodium, and serum levels of IgG were lower in the 12H group than those in the 24H group. The risk score at diagnosis was higher in the 12H group than in the 24H group.

**Table 1:** Baseline demographics and clinical characteristics

	12H group (*n* = 19)	24H group (*n* = 20)	*p* value
Age, month	25 [2–75]	32 [7–118]	0.196
Men	11 (57.9)	11 (55.0)	1.00
Days of illness at enrollment	5 [4–7]	5 [4–6]	0.915
Risk score	4 [0–7]	1 [0–6]	0.022
Laboratory data			
White blood cell count, 10^9^/L	12.4 ± 3.4	12.4 ± 4.0	0.993
Neutrophils, %	71.2 ± 13.3	67.2 ± 15.6	0.403
Platelet count, 10^10^/L	31.1 ± 7.3	36.2 ± 9.3	0.067
Albumin, g/L	3.3 ± 0.4	3.5 ± 0.4	0.111
Creatinine, mg/dL	0.27 ± 0.06	0.27 ± 0.07	0.664
Aspartate aminotransferase, U/L	99 ± 159	38 ± 21	0.092
Sodium, mEq/L	133 ± 3	137 ± 3	< 0.01
C-reactive protein, mg/dL	7.3 ± 3.9	6.4 ± 5.9	0.564
IgG, mg/dL	624 ± 155	754 ± 167	0.021

Data are median [range], number (percentage), or mean ± SD

IVIG indicates intravenous immunoglobulin

IgG indicates immunoglobulin G

12H indicates 12 h

24H indicates 24 h

Because there were statistical differences in risk score, sodium, and IgG level before treatment between the groups, we performed post-hoc analysis. Multivariate analysis using a linear regression model of fever duration after IVIG treatment was performed with the adoption of month, risk score, sodium, and IgG level as explanatory variables. This analysis revealed that only risk score affected it; that is, the fever duration was longer when the risk score was higher (Estimated value: 11.06, 95% CI 5.12–17.00, *p* < 0.01).

### Outcomes

Table 2 presents the results of the primary and secondary outcomes. There was no difference in median fever duration after initiation of IVIG between the two groups (median 21 h.

**Table 2:** Duration of fever, incidence of additional treatment, and coronary artery outcomes

	12H group (*n* = 19)	24H group (*n* = 20)	*p* value
Duration of fever after treatment initiation, h	21.0 [5–130]	21.5 [3–144]	0.325
Non-responder of initial IVIG, n	7 (36.8)	6 (30.0)	0.741
Total dose of IVIG, g/kg	2 [2–6]	2 [2–6]	0.623
Third-line treatment, n	4 (21.1)	1 (5.0)	0.182
Coronary artery abnormalities, n			
Day 0	1	0	0.487
Day 7	1	0	0.487
Day 30	0	0	1.000

Data are median [range], or number (percentage)

IVIG indicates intravenous immunoglobulin

12H indicates 12 h. 24H indicates 24 h

[IQR 16–74] vs. 21.5 h [6–46.5], *p* = 0.325). When limited to initial IVIG responders, 12H group (*n* = 13) showed a longer duration of fever than that of the 24H group (*n* = 15); however, there was no statistical significance (17.5 h [IQR 13–19.5] vs 11.5 h [6–21.75], *p* = 0.424). In addition, the percentage of patients who required additional IVIG treatment did not differ markedly between the two groups (36.8 vs. 30.0%; risk ratio 1.23; 95% CI 0.504–2.995; *p* = 0.741). There were no differences in the total dose of IVIG treatment in this study. The percentage of patients requiring third-line treatment was rather high in the 12H group, but there was no statistically difference between the two groups (21.1 vs. 5.0%; risk ratio 4.21; 95% CI 0.516–34.364; *p* = 0.182). Multivariate logistic regression was performed to identify potential associations with third-line treatment. This revealed that only risk score, not infusion speed, was associated with the need of third-line treatment (odds ratio 2.09, 95% CI 1.07–4.10, *p* = 0.0318).

Transient small coronary aneurysm occurred in two patients in the 12H group. One patient presented with a CAA at study enrollment, which regressed to normal size on day 7 after initial treatment. This patient required 4 g/kg of IVIG. Another patient presented with a CAA at day 7 even though he responded to the initial IVIG. His CAA regressed to normal size at day 30.

There was no difference in the laboratory data before and after the initial IVIG treatment, except for serum concentrations of sodium and serum IgG levels (Table 3). Serum sodium concentration in the 12H group increased from day 0 to day 2. In contrast, in the 24H group, sodium levels did not markedly change before and after the IVIG treatment. This finding may be due to differences in baseline values. Serum IgG levels were increased at day 2 in both groups, but the value was lower in the 12H group, which is expected to have an earlier peak of serum IgG level and faster washout compared to the 24H group.

**Table 3:** Laboratory data

		Day 0	Day 2	Day 7	*p* value
White blood cell count, 10^9^/L	12H	12.4 ± 3.4	8.6 ± 3.9	9.1 ± 4.0	0.585
	24H	12.4 ± 4.0	7.5 ± 3.7	7.9 ± 2.8	
Neutrophils, %	12H	71.2 ± 13.3	46.1 ± 18.4	40.8 ± 12.6	0.732
	24H	67.2 ± 15.6	44.6 ± 19.2	40.2 ± 15.2	
Platelet count, 10^10^/L	12H	31.1 ± 7.3	32.3 ± 10.8	54.9 ± 18.2	0.884
	24H	36.2 ± 9.3	38.3 ± 11.5	58.6 ± 14.9	
Albumin, g/L	12H	3.3 ± 0.4	2.7 ± 0.4	3.4 ± 0.6	0.471
	24H	3.5 ± 0.4	3.0 ± 0.4	3.6 ± 0.5	
Creatinine, mg/dL	12H	0.27 ± 0.06	0.24 ± 0.04	0.25 ± 0.05	0.645
	24H	0.27 ± 0.07	0.26 ± 0.07	0.26 ± 0.06	
Aspartate aminotransferase, U/L	12H	100 ± 159	56 ± 92	39 ± 19	0.186
	24H	38 ± 21	34 ± 13	37 ± 11	
Sodium, mEq/L	12H	133 ± 3	137 ± 2	138 ± 2	< 0.01
	24H	137 ± 3	138 ± 3	138 ± 2	
C-reactive protein, mg/dL	12H	7.3 ± 3.9	5.2 ± 3.5	0.7 ± 0.9	0.509
	24H	6.4 ± 3.9	4.0 ± 5.4	0.8 ± 1.6	
IgG, mg/dL	12H	624 ± 155	2414 ± 248	–	< 0.01
	24H	754 ± 167	3037 ± 648	–	

The value of serum IgG at day 7 was excluded because the value might change depending on whether the additional IVIG was given or not

Data are mean ± SD

IgG indicates immunoglobulin G

12H indicates 12 h. 24H indicates 24 h

Adverse events were observed in two cases. Both cases were in the 12H group. In the first case, the level of aspartate aminotransferase transiently increased. In the other case, vomiting was observed after IVIG infusion; therefore, the infusion speed was reduced. No serious adverse events were observed.

## Discussion

This is the first randomized controlled trial to allocate the administration times of 2 g/kg IVIG to 12 h and 24 to evaluate whether the infusion speed of IVIG affects its efficacy and safety in the acute phase of KD patients. There were no significant differences in fever duration after the initial IVIG treatment, incidence of CAAs, or serious adverse events between the two groups.

Oda et al. compared the treatment effectiveness of 5 and 10% immunoglobulin for the acute phase of KD [9]. They reported that 10% immunoglobulin required one-half the infusion time as that of 5% immunoglobulin, and the fever duration in the 10% group was shorter than that in the 5% group (10 vs. 13 h, respectively). However, the response rate of the initial IVIG treatment and the incidence of CAAs did not differ between the two groups. Their findings suggest that rapid infusion facilitates abatement of systemic inflammation and faster defervescence in the acute phase of KD. On the other hand, our results did not reveal an association between rapid infusion and faster defervescence using a 5% immunoglobulin preparation. In addition, there was no improved response rate to the initial IVIG treatment using a 12-h administration time. One of the reasons for our results may be the patient’s background. In the 12H group, serum sodium and IgG level before initial treatment were lower than those in the 24H group. Since a lower sodium concentration correlate with a greater severity of vasculitis in KD [10], these findings resulted in a higher risk score for IVIG non-responders in the 12H group [7]. Furthermore, a lower serum IgG concentration was reportedly associated with resistance to IVIG treatment [8]. Therefore, patients in the 12H group might have more severe vasculitis than those in the 24H group, and it is conceivable that the difference in severity between the two groups affected the duration of fever.

The additional IVIG administration and the total quantity of IVIG administered did not differ between the two groups. The patients in the 12H group tended to require third-line treatment, whereas IVIG treatment alone tended to effectively treat fever in the 24H group. Since the risk score in the 12H group was higher than that in the 24H group, the incidence of third-line treatment could have been affected by the severity of KD in the 12H group. Therefore, even though additional treatments are required in the 12H group, the superiority of the 24 h infusion over the 12 h infusion is not confirmed in terms of therapeutic efficacy.

No previous studies have examined the association between IVIG infusion speed and the efficacy and change in serum IgG levels in KD. In the current study, the variation in serum IgG levels after the initial IVIG administration was significantly different between the two groups. One possible reason is that the 12H group received IgG earlier, so serum levels fell more quickly than those in the 24H group. The other is that the serum IgG level in patients with KD is presumed to be associated with increased vascular leakage due to vasculitis. Patients with severe KD show lower levels of serum IgG, which is related to resistance to IVIG treatment and the incidence of CAAs [11–13]. Therefore, it is possible that our results––lower serum levels of IgG in the 12H group––only reflected the severity of the disease.

Although the mechanism of action of IVIG treatment in KD has not been clarified, immunoglobulins likely act through many different pathways [14]. Single high-dose IVIG can significantly reduce the incidence of CAAs [3, 4, 15], and IVIG may preferentially elevate serum IgG levels to suppress vasculitis in the acute phase of KD. A shorter infusion of IVIG has some advantages. The reduction in administration time relieves the load on medical staff, shortens the patients’ hospital stay, and allows for earlier determination of efficacy and no delay in additional treatments. On the other hand, even if serum IgG levels increase quickly, the rapid disappearance of IgG from the body will not control the disease, resulting in the need for additional treatment. The slower elevation of serum IgG level by 24 h infusion might maintain a longer-term anti-inflammatory effect.

Although adverse events were observed in the 12H group, neither case was serious. In one case, liver dysfunction was observed during the protocol. The liver enzyme level decreased when changing aspirin to dipyridamole; therefore, this side effect did not appear to be related to the IVIG infusion speed. In the other case, the patient vomited during IVIG administration at infusion rate of 12 h. Slowing down the IVIG infusion rate decreased the patient’s nausea, and additional treatment was not required. Although nausea and vomiting are already known as side effects of IVIG, which occur during the first 30 min of the infusion in many cases [16], it is possible that rapid administration of IVIG may increase the incidence of such side effects. No serious side effects were observed in the 12H group, therefore, it is thought that there is an advantage in reducing the time required for medical care by administering the drug in a short time.

This study has some limitations. First, the current study included a small sample size. We were unable to enroll the required number of patients because the gamma globulin preparation specified for this study was temporarily unavailable and the study had to be terminated in 2019. Second, randomization adjusted by age and sex caused a difference in risk score between the two groups. Because the severity of KD would affect the treatment responsiveness, the effect estimates would be biased due to randomization failure. Third, echocardiography was performed by pediatricians who were not blinded to the assignment at each institution; as data were not masked for clinical information, there is a possibly of biased measurement of coronary arteries. In addition, the generalizability of our results has not been verified; therefore, further study is needed to verify the external validity.

## Conclusions

This was the first randomized controlled trial to evaluate the efficacy and safety of two different infusion speeds (double speed and standard speed) of IVIG in the acute phase of KD. The results showed no difference in the fever duration between the 12 h and 24 h IVIG treatments and a low incidence of adverse events in both groups. Although differences in disease severity were suggested between the two groups, IVIG administered over 12 h was similar to that over 24 h with regard to efficacy and safety.

## Data Availability

No supporting datasets are available because participants of this study did not agree for their data to be shared publicly.

## Abbreviations

IVIG: intravenous immunoglobulin
KD: Kawasaki disease
CAAs: coronary artery abnormalities
IgG: immunoglobulin G
12H: 12 h
24H: 24 h
